# Review on the Vascularization of Organoids and Organoids-on-a-**C**hip

**DOI:** 10.3389/fbioe.2021.637048

**Published:** 2021-04-12

**Authors:** Xingli Zhao, Zilu Xu, Lang Xiao, Tuo Shi, Haoran Xiao, Yeqin Wang, Yanzhao Li, Fangchao Xue, Wen Zeng

**Affiliations:** ^1^Department of Cell Biology, Third Military Medical University, Chongqing, China; ^2^State Key Laboratory of Trauma, Burn and Combined Injury, Chongqing, China; ^3^Department of Neurology, Southwest Hospital, Third Military Medical University, Chongqing, China

**Keywords:** organoid, organiods-on-a-chip, vascularization, advanced printing methods, micro-environment

## Abstract

The use of human cells for the construction of 3D organ models *in vitro* based on cell self-assembly and engineering design has recently increased in popularity in the field of biological science. Although the organoids are able to simulate the structures and functions of organs *in vitro*, the 3D models have difficulty in forming a complex vascular network that can recreate the interaction between tissue and vascular systems. Therefore, organoids are unable to survive, due to the lack of oxygen and nutrients, as well as the accumulation of metabolic waste. Organoids-on-a-chip provides a more controllable and favorable design platform for co-culture of different cells and tissue types in organoid systems, overcoming some of the limitations present in organoid culture. However, the majority of them has vascular networks that are not adequately elaborate to simulate signal communications between bionic microenvironment (e.g., fluid shear force) and multiple organs. Here, we will review the technological progress of the vascularization in organoids and organoids-on-a-chip and the development of intravital 3D and 4D bioprinting as a new way for vascularization, which can aid in further study on tissue or organ development, disease research and regenerative medicine.

## Introduction

Recent years have seen wide development of organoids and organoids-on-a-chip, as they are important in the imitation of the structure and function of human organs. At present, vascularization attempts of organoids and organoids-on-a-chip have attracted attention. Thick organ tissue requires an abundant network of micro-vascular vessels to provide oxygen and nutrients, as well as handle the discharge of metabolic waste. Specifically for organoids-on-a-chip, vascularization helps us observe the bio-chemical reactions and transport of substances in vascular tissue. In recent years, the literature has focused on the *in vitro* regeneration of angiogenesis. At the same time, the micro-flow control system successfully simulates the precise regulation of the tissue micro-environment in many aspects and provides biochemical and mechanical shear force as a method for *in vitro* vascular network construction. Therefore, we aim to establish a macro-micro bridge by reviewing the current research on micro-flow control technology, providing ideas for the precise regulation of the complex tissue structure model of *in vitro* organ reshaping and exploring the interaction of vascularization in various tissues. These concepts are of great significance to the next generation of vascular tissue engineering and the development of regenerative medicine.

## Vascularization of Organoids

For the organoids having been constructed, the mainly limitation of achieving completely functional organoids like *in vivo* is the lack of rational tissue size. The main cause of growth arrest or cell death of all organoids in tissue engineering is the lack of adequate oxygen and nutrient supply. The maturation of organoids is influenced by the limitation of nutritional supply (Lancaster and Knoblich, 2014). The transport of oxygen and nutrients *in vivo* to tissue cells through diffusion is limited within a few hundred microns of capillaries (Yin et al., 2016). Additionally, the removal of cellular metabolic waste is also essential for the survival of cells (Muschler et al., 2004). Therefore, it is necessary for the successful development of organoids to remodel functional vessel networks for most organs with high metabolism, such as the heart, liver, kidney, and brain (Auger et al., 2013).

The approaches for organoid vascularization can be sorted into two categories: *in vitro* and *in vivo* vascularization. *In vitro* vascularization is obtained by co-culture with vascular cells or tissue engineering (Mansour et al., 2018). The strategies for *in vitro* vascularization can be sorted into templating and self-organizing methods (Nashimoto et al., 2017). Templating methods include hydrogel molding by needles, sacrificial molding, assembly of patterned hydrogel slabs and bio-printing (Nashimoto et al., 2017). Endothelial cells are co-cultured with supporting cells in the self-organizing method (Nashimoto et al., 2017) and neo-angiogenesis is induced by angiogenic growth factors, which can also promote cell self-assembly by gradient (Yin et al., 2016). *In vivo* vascularization is achieved by transplanting organoid models built *in vitro* into a host. The following paragraphs illustrate representative studies for each type of organoid vascularization approach.

### Vascularization *in vitro*

#### Templating Method

This method has been applied into different strategies of realizing vascularization of heart tissue.

**FIGURE 1:**
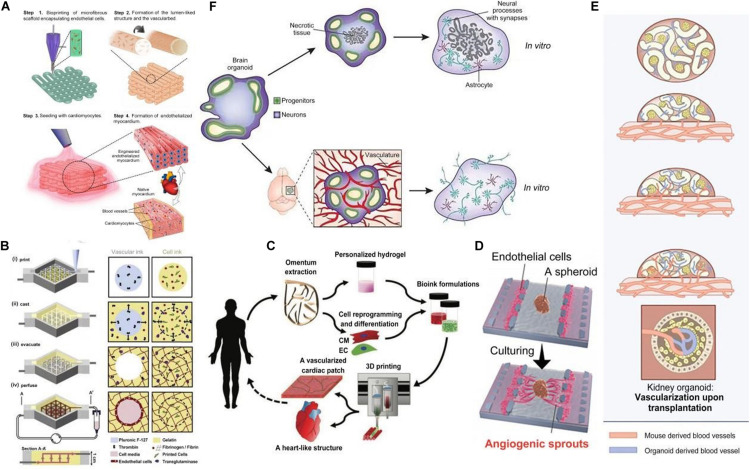
Vascularization of organoids in various methods. **(A)** The procedure of endothelialized myocardium fabrication using the 3D bio-printing strategy. **(B)** Schematic illustration of the tissue manufacturing process. **(C)** Concept schematic of printing a full, thick vascularized tissue in one step. **(D)** Overview of microfluidic device use for angiogenic sprout inducing in a human lung fibroblast (hLFs) spheroid. Reprinted with permission from Nashimoto et al. (2017). **(E)** Upon transplantation, host-derived vascular networks invaded the hPSC-derived kidney organoids and connected to the organoid-derived plexus. **(F)** Brain organoids grown *in vitro* and transplanted into the mouse cortex.

The first strategy is printing the endothelial cells (ECs) without the parenchymal tissue. This method is enabled with the use of a composite bio-ink encapsulating ECs (Zhang Y. S. et al., 2016) (Figure 1A). A micro-fibrous scaffold can be bio-printed using this bio-ink, ECs can be directly bio-printed within scaffolds and then gradually migrate toward the periphery of the microfibers to form a layer of confluent endothelium. Along with controlled anisotropy (achieved by capability of their technique to bioprint 3D microfibrous scaffolds with anisotropic arrangements), cardiomyocytes are seeded into the interstitial space of the endothelialized scaffold to generate an aligned myocardium layer capable of spontaneous and synchronous contraction.

The second strategy is printing ECs along with surrounding tissues followed by external perfusion of ECs. A technique for hydrogel construct vascularization that confirmed endothelial monolayer formation within the fabricated channels has been reported by Bertassoni et al. (2014). They described a 3D micro-molding technique that utilized bio-printed agarose template fibers to fabricate micro-channel networks with various architectural features within photo-cross-linkable hydrogel constructs and successfully embed micro-channels inside hydrogels. The bio-printed templates can then be easily removed to form fully perfusable networks without any template dissolution. A method for bio-printing 3D cell-laden, vascularized tissues that exceed 1cm in thickness and can be perfused on a chip for long time periods (>6 weeks) can be used to improve perfusion (Figure 1B). A technique for integrated parenchyma, stroma and endothelium into a single thick tissue by co-printing multiple inks composed of hMSCs (human mesenchymal stem cells) and human neonatal dermal fibroblasts (hNDFs) within a customized extracellular matrix alongside embedded vasculature, which was subsequently lined with HUVECs (human umbilical vein endothelial cells) has been reported by Kolesky et al. (2016). The vascularized tissues were then perfused with growth factors. Miller et al. (2012) reported a general approach for rapid construction of such networks. They printed rigid 3D filament networks of carbohydrate glass and used them as a cyto-compatible sacrificial template in engineered tissues to generate cylindrical networks that could be lined with ECs and perfused with blood under high-pressure pulsatile flow. This simple vascular casting approach is compatible with a wide variety of cell types, synthetic and natural extracellular matrices and cross-linking strategies.

The third is printing a full, thick, vascularized tissue in one step. Noor et al. (2019) reported 3D printing techniques using personalized hydrogel as a bio-ink and printing a full, thick, vascularized tissue in one step (Figure 1C). Omentum tissue was isolated from the patient and cells were separated from the matrix. The matrix was processed into a personalized, thermo-responsive hydrogel. The cells were reprogrammed and encapsulated within the hydrogel to generate the bio-inks used for printing. The bio-inks were then used to print vascularized tissues. It was demonstrated that the personalized hydrogel can be used to print volumetric, freestanding, cellular structures, including whole hearts and their major blood vessels.

#### Self-Organizing Method

The advantage of templating is that it is usable immediately after manufacture (Nashimoto et al., 2017). However, the non-dynamic of vessel structure hinders the functional vessel formation, therefore, cells cannot dynamically adapt to the environment of the surrounding tissue, during co-culturing (Nashimoto et al., 2017). By contrast, *in vitro* vascular network established by the self-organizing method resembles angiogenesis, morphology and permeability *in vivo* more closely (Nashimoto et al., 2017).

Nashimoto et al. (2017) used a microfluidic device to induce angiogenic sprouts in a human lung fibroblast (hLFs) spheroid, which led to perfusable self-assembled vascular networks (Figure 1D). The spheroid was prepared from co-cultured HUVECs and hLFs, in which vessel-like structures would form. T he spheroid was then introduced to the spheroid well in the microfluidic device and HUVECs were seeded in the left and right channels of that well. The soluble angiogenic factors secreted by HLFs induced the formation of angiogenic sprouts toward the spheroid. These angiogenic sprouts grew into the perfusable vascular networks supporting active transport, which could anastomose to the previous vessel-like structures in the spheroid. Additionally, these vascular networks could transport nutrients and oxygen to cells in the spheroid and dispose of the metabolic product, which is similar to the *in vivo* physiological functions. The perfusable vessel model constructed by this approach subsequently led to culturing conditions the in spheroid that were more beneficial, which provides an effective model for long-term *in vitro* tissue culture.

A strategy for vascularization of brain organoids includes inducing the formation of blood vessel-like structures by vascular endothelial growth factor (VEGF) *in vitro*. Ham et al. (2020) applied this strategy for *in vitro* the formation of blood vessel-like structures in cerebral organoids. The results indicated that VEGF enhanced the differentiation of vascular ECs without reducing neuronal markers in the embryonic bodies (EBs), which then successfully developed into cerebral organoids with open-circle vascular structures expressing characteristic of the blood–brain barrier (BBB). Therefore, VEGF treatment can be used to generate vessel-like structures with mature BBB characteristics in cerebral organoids *in vitro.*

Another strategy for brain organoid vascularization involves achieving revascularization from the outside surrounding matrix. Pham et al. (2018) modeled the developmental peri-neural vascular plexus by coating the whole-brain organoid with Matrigel-embedded ECs. They achieved revascularization from the outside surrounding matrix, as opposed to direct injection into the center of the organoid. Induced pluripotent stem cells (iPSCs) were grown into whole-brain organoids. Simultaneously, iPSCs from the same body were differentiated into ECs. The organoid was then re-embedded in Matrigel with 250.000 ECs. Coating of brain organoids with ECs led to robust vascularization of the organoid. Vascularized organoids were grown *in vitro* and then transplanted into immunodeficient mice, to found that blood vessels did not stay on the periphery of the organoid but instead penetrated its center *in vivo*.

The approaches of vascularization *in vitro* can fully control over the growth of vascular network and immediate functionality. However, it still needs to be improved to be more viable and changeable because of the incapability to adapt to the real-time change of organoids.

### Vascularization *in vivo*

Though current progress in microfluidic technology has demonstrated the feasibility of tissue angiogenesis, these methods will disturb the self-organizing structure in organoids (Lancaster, 2018). So far, transplanting organoids into hosts has been the only way to achieve tissue vascularization with complete function (Grebenyuk and Ranga, 2019). When the organoid is transplanted into hosts, the vascularization process mimics the native angiogenesis that occurs in the human body (Lancaster, 2018). Thus, *in vivo* vascularization by transplantation will develop organoids with complete function more efficiently and be more beneficial to the survival of organoids.

Van den Berg et al. (2018) used the approach of transplantation and described the vascularization of kidney organoids (Figure 1E). Human pluripotent stem cell (hPSC)-derived kidney organoids were transplanted under the kidney capsule of host mice. Then they observed that the host-derived vascular networks invaded the developing glomerular structures in the organoids and actively connected to the human-derived plexus. In addition, they confirmed that the organoids in hosts performed more integrated process of development and maturation, in comparison with the non-transplanted organoids. However, unsolved defects still exist in vascular networks generated in hosts, for instance the functional maturity of blood vessels in kidney organoids is not on part with that of kidneys in human (Koning et al., 2020).

Takebe et al. (2014) used the same approach to develop vascularized liver organoids. They cultivated human iPSCs-derived hepatic endoderm cells (iPSCs-HEs), HUVECs and hMSCs, which self-organized to liver buds (iPSCs-LBs). Then they transplanted the iPSCs-LBs under the cranial window in mice. Results showed that human-derived vessels connected to the host vessels and formed unobstructed conduits which could deliver nutrients and oxygen (Takebe et al., 2013). This work shows that transplantation of *in vitro* cultured iPSCs-LBs into a host could develop vascularized liver organoids with functional tissue architecture (Takebe et al., 2013).

Mansour et al. (2018) reported on the generation of vascularized brain organoids after transplantation into the mouse brain, at the Society for Neuroscience Meeting 2017. This research focused on the issue of vascularization by transplanting cerebral organoids onto a vascular bed in the cortex of an adult mouse. They showed that intra-cerebral transplantation of brain organoids in mice resulted in impressive growth of blood vessels into the human tissue, with clear benefits for cell survival and maturation compared with organoids kept *in vitro* (Figure 1F).

Currently, the main challenge of achieving completely functionalized organoid is achieving spontaneous perfusing capability of blood vessels. The approaches of vascularizing organoids both *in vitro* and *in vivo* have not achieved this goal. Future researches of vascularized organoids will focus on the angiogenesis principles to induce viable and functional vasculature. With the advance of tissue engineering technologies and the improvement of existing approaches, the barriers of vascularizing organoids will be cleared up, which will promote the substantial production of implantable organoids with fully functionalized vessels.

## Vascularization of Organoids-On-A-Chip

### The Importance of Organoids-on-a-Chip

Traditional *in vitro* cell culture techniques frequently use culture bottles, culture dishes, etc. as a living environment for cells. This 2D culture method lacks the complex living environment the cells grow in *in vivo* and cannot stimulate the cells by specific physical and chemical factors, such as biochemic concentration gradient, fluid shear force and mechanical stress. Through this method, the cells cannot achieve the level of self-assembly and truly restore the specific physiological function of the organ prototype (Bhatia and Ingber, 2014).

The gold standard for biological testing and animal models also has certain defects. Due to differences in biological metabolism and immune function between animals and humans, there still remains uncertainty such as medical diagnoses (Bhushan et al., 2013). Therefore, development of an effective model that simulates the human body is particularly important.

Organoids-on-a-chip is an effective alternative solution. During the construction of organoids-on-a-chip, it is necessary to consider the anatomical structure of the target organ and restore its basic physiological and characteristic structure. In this way, we are able to design the 3D mechanical and biochemical environment that corresponds to the target organ, according to the growth characteristics of the cell and use micro-processing technology (such as soft lithology) to construct the model necessary for cell growth (Park et al., 2019). The model is widely used in the study of organ function (Occhetta et al., 2018), disease modeling (Mazio et al., 2018) and pharmacological modeling (Sung et al., 2014). Blood vessels are important in connecting organs and realizing material exchange between organs. Additionally, micro-vascular networks maintain metabolism and the stability of the tissue micro-environment. The vascularization of organoids-on-a-chip provides us with a good platform to explore the physiological barrier role of blood vessels (Tarbell, 2010), the pathological study of blood vessels by blood flow shear force (Zheng et al., 2012), vascular regeneration (Zheng et al., 2012) and substance transport between tissues (Skardal et al., 2017).

### Regulation in Micro-Environment

Micro-environment conditions determine the growth and development of cells in organoids-on-a-chip, among which micro-flow models, embedded hydrogels and fluid shear forces are important conditions that affect the vascularization of organ chips.

In micro-flow models, cell adhesion of biomass formed by polymers, such as polydimethylsiloxane (PDMS), is mostly poor. Cell adhesion is promoted by coating its surface with a layer of fibrin or hydrogel material (Taylor et al., 2005; Park et al., 2006). In addition, the size of channel has an important effect on the growth of vascular ECs. ECs extend in all directions in channels of 250–500 μm. Reduction of the inner diameter of the channels, causes almost all ECs to extend along the axial direction of the flow path, especially in the channels ranging from 10–20 μm (Greca et al., 2018), which are similar to the size of capillaries (i.e., less than 10 μm) *in vivo*. However, due to the accuracy of the manufacturing process, the formation of micro-vessels *in vitro* is usually greater than 50 μm (Haase and Kamm, 2017). The construction of small lumen plays an important role in the simulation of blood microcirculation.

The properties of hydrogels have a noteworthy influence on the formation process and function of microvascular networks. In hard hydrogels, the resulting microvascular network tends to have smaller channel diameters, thus limiting the migration of ECs (Chung et al., 2009). On the other hand, although the hydrogel itself has pores, they are mostly nanoscale, which makes nutrient and cell metabolic waste hard to discharge inside the hydrogel, hinders the function of cells and is not conducive to the fusion of body tissue. Ying et al. (2020) Developed macro-micro-nano-porous cell-laden gelatin methacryloyl (GelMA) hydrogel constructs by 3D extrusion bio-printing technology. Because hydrogels have better permeability than PDMS, plastics, silicon and many other substrate materials. HeYong team (Nie et al., 2018) develops some new methods in manufacturing micro-flow control chip based on a twice cross-linking hydrogel bulk. Hydrogel chips with good mechanical and biological properties were developed through cross-linking the commonly used alginate, gelatin and GelMA. The chip was very similar to natural extracellular matrix (ECM) in both water content and cytokine diffusivity and can be made into vascular chips in order to simulate disease models of blood vessels. The addition of specific acellular extracellular matrix into hydrogels as bio-inks has been reported to also promote angiogenesis (Zheng et al., 2020). Hydrogels with high biocompatibility are mature platforms for 3D cell culture. Appropriate hardness and pores are conducive to the growth of microvessels in hydrogels and the diffusion of various pro-vascular growth factors in hydrogels.

Mechanical stress affects the shape and function of the resulting blood vessels. Hemodynamic disorders are also easy to cause thrombus and atherosclerosis and induce inflammation. In increasingly fast blood flow, the vascular barrier function is the same as the one in the human body. Additionally, cyclic adenosine monophosphate (cAMP) in vascular ECs increases, under fast blood flow, improving the selectivity of the vascular barrier and the proliferation of ECs (Price et al., 2010). These findings provide good starting ground for building artificial blood vessels *in vitro*. Moreover, Vickerman’s studies have found that high vascular shear force is important for the formation of new blood vessels (Vickerman et al., 2008; Vickerman and Kamm, 2012). Appropriate blood flow is essential to simulate organ function when constructing organ microarray. The existing problem is that vascular ECs are difficult to arrange evenly and neatly in the constructed vascular lumen. This phenomenon is more prominent in the complex vascular network structure. The perfusion blood shear force can cause damage to the vascular ECs attached to the lumen and mediate the occurrence of subsequent vascular diseases. On the other hand, blood vessels themselves have tension, and the dilation of blood vessels has a great influence on the growth of ECs, but these have been ignored in most of the construction of vascularized organ chips. At the same time, it also puts forward higher requirements for the selection of vascular construction materials.

### Application of Vascularization in Organoids-on-a-Chip

Development of organoids-on-a-chip has recently been seen significant growth. Several functions in the target organs have been successfully reproduced, many of which are closely related to the vascularization of organoids-on-a-chip. Functional microvascular structure provides a model for the study of complex vascular phenomena *in vitro*.

#### Lung-on-a-Chip

**FIGURE 2:**
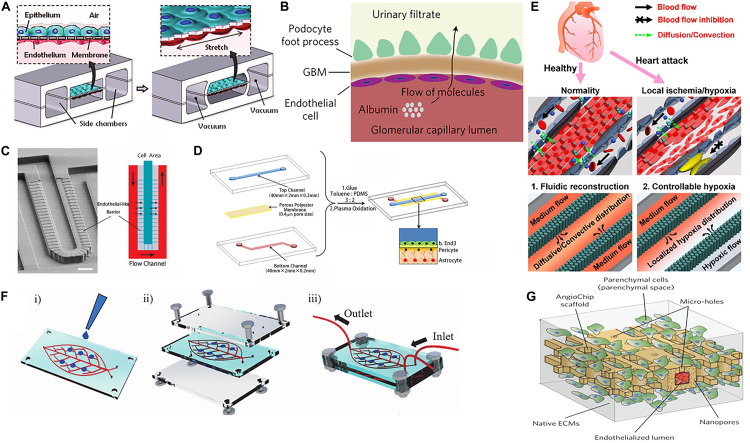
Vascularization of organoids using various methods. **(A)** The alveolar epithelial cells and the vascular endothelial cells with flexible PDMS membrane form an alveolar-capillary barrier. Adapted from Huh et al. (2010). Reprinted with permission from AAAS. **(B)** Schematic representation of glomerular capillary wall with podocytes and endothelial cells that form a filtration barrier. **(C)** SEM micrograph and structure diagram of microfluidic chip simulates the hepatic sinusoid model. **(D)** Endothelial cells, pericytes, and astrocytes construct the blood–brain barrier (BBB). **(E)** The microfluidic device for studying controllable myocardial hypoxia and for myocardial fluidic microenvironment mimicking. **(F)** Human-on-leaf-chip simulates the complex vascular network structure of the human body. **(G)** AngioChip with branching interconnected lumen composed of POMaC and biological components.

Lung-on-a-chip was the first organoids-on-a-chip to be developed (Huh et al., 2007, 2010, 2012) (Figure 2A). A porous PDMS membrane covers the middle area of the chip. The alveolar epithelial cells are attached to the upper surface of the membrane in a gas channel and the vascular ECs are attached to the lower surface of the membrane in a blood channel. Two side channels connected to the vacuum pump on both left and right sides of the channel cause the PDMS membrane to deform under vacuum conditions to simulate the expansion and contraction of the alveolar wall during breathing. This model is a good simulation of the alveolar-capillary barrier. The micro-porous pathways of this model can be used to simulate pulmonary edema (Huh et al., 2012), as well as chronic obstructive pulmonary disease (COPD) (Benam et al., 2016) caused by viral or bacterial infections. The lung-on-a-chip has been used to explore disease models between vascular ECs and alveolar epithelial cells and findings have revealed that periodic respiratory rhythms play an important role in the development of lung diseases.

#### Kidney-on-a-Chip

Early studies using kidney-on-a-chip have simulated glomerulus, proximal convoluted tubule and proximal convoluted tubule in the structure of kidney units, achieving blood filtration and re-absorption of initial urine (Weinberg et al., 2008). Furthermore, the introduction of fluid shear force has been reported to improve the re-absorption capacity of albumin and glucose of kidney-on-a-chip (Duan et al., 2008; Jang and Suh, 2010). Mu et al. (2013) stitched together two hydrogels through hydrogel bonding technology, forming two parallel 3D micro-vein networks. Madin-Darby Canine Kidney (MDCK) cells and HUVEC cells were cultured in these networks respectively, so that the formation of the kidney hydrogel chip simulated the passive diffusion of kidney units. This was an effective way to increase the diversity and complexity of kidney-on-a-chip. The majority of previous studies have focused on renal tubular epithelial cells, however, the re-emergence of the function of the kidney filter barrier is gradually gaining popularity using the combination of stem cell technology and regenerative medicine. On both sides of the porous PDMS, hiPS-cell-derived podocytes and primary human glomerular ECs are attached, simulating the urinary and capillary compartments of the glomerulus respectively. The high retention rate of albumin and the high permeability of inulin prove the successful construction of kidney filter barrier *in vitro* (Musah et al., 2017) (Figure 2B). These findings can be used in the exploration of the physiological and pathological function of the kidneys.

#### Blood–Brain Barrier-on-a-Chip

The BBB is a highly selective barrier structure that separates the brain and central nervous system from blood circulation. The BBB is a network of interacting blood vessels, pericytes and astrocytes that provides oxygen and nutrients to the brain. It is very important to maintain the physiological activities of the central nervous system and the homeostasis of the microenvironment in the brain. It also has a guiding significance for how drugs act on the central system through the BBB. Wang et al. (2016) Further establish BBB model by co-culture of cerebral microvascular ECs and rat primary astrocytes on both sides of the porous membrane (Figure 2D). The results show that the activity of the cells are still in good condition for 21 days after construction. Each side of the BBB forms a monolayer of cells, simulating the BBB permeability to a certain extent. In the following research, key elements such as the inclusion of more types of brain cells, extracellular matrix and mechanical fluid conditions will be the direction for the functions of brain research. The BBB-on-a-chip can be constructed in many ways, but the vascular bed in the brain vessels is much tighter than the blood vessels in other peripheral organs. It requires us to use some special indicators to evaluate its function, such as TEER, small molecule permeability test, etc. (Oddo et al., 2019). The development of BBB chips provides an innovative approach for brain-related research, including modeling of neurodegenerative diseases and high-throughput drug screening.

Other single-organ chips such as liver chip (Lee et al., 2007; Kang et al., 2015) (Figure 2C), heart chip (Grosberg et al., 2011; Ren et al., 2013; Shao et al., 2016) (Figure 2E) and so on have also attracted attention with significant development. Several studies on organoids-on-a-chip have also focused in the observation of the drug responses in tissues and in the detection of the toxic and side-effects of target drugs on tissue chips *in vitro*. With the development of organoids-on-a-chip technology, the “multi-organ chip” (Oleaga et al., 2016), which contains more than ten kinds of organs, is gaining popularity. The chip channels provide a platform for the connection between the organs on the chip, which can be used to construct the ADME model to detect the interaction of drug effects between multiple tissues (Oleaga et al., 2016; Skardal et al., 2017) and the effect of tumor-on-a-chip on other tissue chips (Skardal et al., 2016; Greca et al., 2018).

Cells and organs establish contact with the peripheral circulatory system by secreting soluble factors and extracellular vesicles. A microfluidic system is used to connect different organoids-on-a-chip to simulate blood perfusion *in vivo* and regulate the culture environment. Vascularization between organs is an important aspect to be realized, as a bridge for intra-organ communication. At the same time, organoids-on-a-chip mostly uses parallel channels (Biselli et al., 2017; Chung et al., 2017) and seldom connects with each other through the vascular structure. This structure is quite different from the multi-stage complex vascular network *in vivo*, which makes providing a real physiological micro-environment (e.g., shear force stimulation) and simulating multi-organ signal communication conduction difficult. Therefore, the emergence of a bio-mimetic vascular leaf chip addresses those limitations (Mao et al., 2020) (Figure 2F). Through artificially edited digital models, photolithography is used to form an embedded multi-scale vascular network chamber, in which vascular ECs are growing. This model reconstructs complex physiological features, similar to those of the blood circulation system in the human body. The 3D vascularized organs in the larger chambers on the chip communicate with each other through the vascular network. It was found that pancreatic tumor cells tended to migrate to bone tissue and interact with bone MSCs. Milica Radisic’s team simulated the vascular network system with poly(octamethylene maleate (anhydride) citrate) (POMaC), which contains microns and nanoscale micro-pores. And they successfully constructed functional and vascularized liver and heart tissue (Zhang B. et al., 2016) (Figure 2G). In the case of multi-organ devices, simple diffusion or convection of soluble factors between different regions on the chip can be realized by means of inter-organ communication through vascularization. The monitoring of each factor in this process can be more conducive to our cognition of some physiological and pathological processes and impose intervention conditions from them. The main challenge in establishing inter-organ linkages is to develop and optimize the medium formulation for each organ. At the same time, reducing the effects of unnecessary metabolic waste from the previous organ on the blood vessels and the next organ also needs to be addressed.

## Advanced Printing Methods

The cutting-edge vascular printing methods that are presently available include extruded 3D printing and the improvement of printing materials and printing methods based on extruded 3D and 4D printing based on projection light-curing printing. At the same time, new applications of extruded 3D printing emerge in the construction of vascularized tissue, vascular-like structure and vascular network structure.

Professor Ali Khademhosseini’s team developed GelMA-based vascularized skin using 3D printing by using GelMA/alginate saline gel containing HUVECs on a porous polyester membrane with a pore diameter of 0.4 μm. This structure is beneficial to the interaction between dermal fibroblasts and ECs and promotes the diffusion of nutrients to form an internal vascular network (Barros et al., 2020). Songwan Jin’s team implemented 3D printing to develop multi-scale hepatic lobules with highly vascularized structure. The hepatic lobular vascular system was realized through the use of coaxial nozzles and sacrificial materials, leading to the adjacent ECs at the lumen or surface to form a cell layer, thus realizing the connection of ECs between the lumen and the surface. Vascular-like structures include trachea, gastrointestinal tract, renal tubule and urinary catheter, which are distributed all over the body and the diameter of the tube ranges from micron to centimeter (Kang et al., 2020). The mechanical strength of micro-tubules constructed by coaxial printing can be improved, through the construction of a new type of mixed high-strength hydrogel 3D printing ink (Liang et al., 2020). The construction of the entire vascular network including macro-vessels and capillaries is based on the hydrogel and the secondary cross-linking encapsulation method of hydrogel has been proposed according to the cross-linking characteristics of the hydrogel. A variety of vascular models with physiological significance can be constructed, such as bifurcated vascular network, spiral vessels and vascular stenosis (Greca et al., 2018).

**FIGURE 3:**
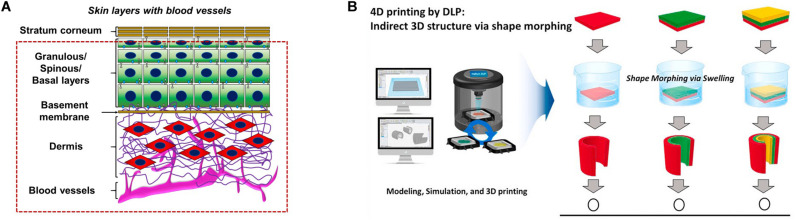
**(A)** Layered printing to achieve vascularized skin. **(B)** The advantage of digital light processing 4D printing is that on the basis of digital light processing 3D printing, one material is used to manufacture structures with multiple curvatures. Reprinted with the permission from Elsevier (Kim et al., 2020; Su et al., 2020). With the permission from Elsevier. Reprinted from Barros et al. (2020) and Kim et al. (2020).

In addition to extruded 3D printing, new conceptual printing methods, such as 4D biological printing, combine “time” as the fourth dimension with 3D printing to simulate the complex dynamics of natural tissue (Figure 3). The 4D printing method is based on projection light-curing printing. Compared with the traditional light-curing printing, it can greatly reduce the printing time, while replacing multiple groups of material components (Kim et al., 2020). Future improvements include printing of microfluidic channels directly on curved surfaces such as the skin, allowing real-time detection of body fluids and bodily functions (Su et al., 2020).

## Conclusion

Developing tissue in organoids requires interaction of a vascular network within the appropriate diffusion distance to enable the exchange of oxygen, nutrients and metabolic substances. Consequently, the phenomenon of grow arrest in organoids can be attributed to the lack of vascularization. According to the categories of organoid vascularization approaches, both *in vitro* and *in vivo*, we respectively demonstrate these methods by commenting on past literature. We discussed the advantages and disadvantages of these approaches and draw a conclusion that only the method of transplantation *in vivo* can lead to angiogenesis similar to that in human. However, none of these methods have achieved a vessel network standard in organoids, the same as that *in vivo*.

The micro-flow control device provides a good platform for building vascular chips *in vitro*, regarding organoids-on-a-chip. By regulating micro-flow control models, hydrogels, fluid shear forces, etc., we can have a clearer understanding of the interaction of blood between vessels and tissues. At present, the technology of vascular regeneration has matured, nevertheless, It requires further improvement of its long term application that maintains good functional activity and simulation of relative changes in the high-volume micro-flow control chip. Simultaneously, the successful realization of vascularization in organoids-on-a-chip can also provide ideas for the realization of organoid vascularization.

## Future Directions

Organoids and organoids-on-a-chip are important means for 3D culture *in vitro*. They overcome many shortcomings of traditional 2D culture and are better in restoring the specific function of organs.

The largest obstacle for organoid maturation is the lack of functional vascularization, this leads to the lack of rational tissue size. The field of organoid vascularization has only recently started developing. And many challenges are on the way of realizing fully functional and spontaneous perfusing capability vascularization. Finding methods of constructing functional vessels in organoids with perfusing capability same as that in humans is the focus of future studies. The strategy based on the developmental principles of vasculature should receive more attention. The engineered vascular tissue should be highly consistent with the vascular development process *in vivo*, both in time and space, in order to form a vascular network with the same functionality. Additionally, new bioengineering approaches are needed to provide long-term cultivation of the organs and efficient mass transfer, while supplying biochemical and physical cues for maturation. For the printing method, advanced technologies to precisely print small-diameter blood vessels should be developed. Establishing a mature system of transplantation methods and cytokine induction is essential for *in vivo* vascularization. With the progress of micro-fabrication technologies and the improvement of previous methods, new methods will emerge in organoid vascularization. The combination of multiple methods may replace the single technical strategy to vascularize organoids. Organoids with fully functional vessels will become real substitute organ models to serve for various fields.

Challenges and problems still exist in the vascularization of organoids-on-a-chip. Due to the limited size of the chip, the implant is embedded on the chip and does not allow adequate space for the formation of a complex network of blood vessels, which may be considered for the expansion of the chip. HUVECs are mostly used as the EC phenotype. However, organ-specific vascular ECs or pluripotent stem cell are recommended for a more realistic restoration of the target organ. In addition, co-cultured cells also have a significant effect on blood vessel growth (Norotte et al., 2009). At present, many vascular organoids-on-a-chip do not apply pericytes to the vascular network, ignoring the important role of the pericytes in regulating and maintaining the growth of blood vessels (Orekhov et al., 2014). In research on therapeutic applications, we can use patient-specific ECs (and support cells) to avoid immune rejection of such implants. This also allows for a more accurate reflection of the function of a particular part. The biggest challenge today remains the precise simulation of the internal environment, such as the shape of the micro-flow model, hydrogel properties, the chemical gradient of cytokines and mechanical stress, which all affect the vascularization of organoids-on-a-chip. Combining organoids-on-a-chip with electronic components will also be a trend. To detect the output, studies have investigated electronic components loaded into organoids-on-a-chip, such as 3D multiwell–multielectrode devices (Eichler et al., 2015), that detect hydrogel and cellular impedances to improve detection of cell toxicity and drug reactions. This also provides an intuitive method for *in vitro* indicator observation. The combination of sensors and organoids-on-a-chip will see further study, however, requirements for the accuracy of sensors increase. For vascularized multi-organoids-on-a-chip, blood vessels acting as a path to connect tissues and loading sensors on the blood vessels can help monitor the overall situation of the chip. The development of non-destructive and real-time methods to characterize the vascular networks in organs on a chip will be the key to the development of the next generation of vascularized organoids-on-a-chip. In addition, further optimization should be made in culture conditions (such as medium composition) to support specific organs and develop vascular networks. The development of a universal medium to ensure that all organs receive essential nutrients and minimize unnecessary toxic reactions is an unresolved issue in the development of a multi-organoids-on-a-chip model.

## Author Contributions

WZ directed the completion of this article. XZ and ZX wrote the main content of this article. TS, HX, and LX collected the information and created pictures for this article. YL, FX, and YW provided advice for this article. All authors contributed to the article and approved the submitted version.

## Conflict of Interest

The authors declare that the research was conducted in the absence of any commercial or financial relationships that could be construed as a potential conflict of interest.
